# Editorial: Feeding Difficulties in Children and Adolescents

**DOI:** 10.3389/fped.2018.00105

**Published:** 2018-05-04

**Authors:** Mauro Fisberg

**Affiliations:** Feeding Difficulties Center, Pensi Institute, Jose Luiz Setubal Foundation, Sabará Children's Hospital, Sao Paulo, Brazil

**Keywords:** childhood, eating disorders, feeding problems, infancy, multidisciplinary communication, phobic disorders, selective intake

Some months ago, together with Frontiers Pediatrics, we decided to launch a new topic of discussion about a common problem in childhood, often addressed in the pediatric consultations around the world: Feeding Difficulties. We expected to open a new field to discuss one of the most important problems in pediatric consultations globally. Informal data from many regions of the world showed that parents are concerned about children's feeding, in almost 50–60% of the cases, and that those numbers increase if the child has a neurodevelopmental problem. One of the initial concerns that we discussed at the launching of the topic, was that the majority of the papers on this theme were related to consequences of gastrointestinal problems and as result of oral motor issues, after hospital discharging. We wanted to show that feeding difficulties do occur in most of the regions around the world, in the so called normal children, as part of normal development. Although more prevalent in children with organic problems or developmental disabilities, this multifactorial disorder also affects children with typical development behavior. Feeding difficulties ranges from errors in interpretation of amounts of food to be given to children to patients that may show several inadequate eating behaviors, like anorexia and hyper neophobia, besides impaired sensorial processing and delayed motor skills development. All of this problems could interact and an organic cause to feeding problems could lead to an important modification of the dynamics of families, transforming it in a behavioral situation (1, Ramos et al).

Empirically, it is estimated that almost 20–60% of the parents are concerned about their children's feeding, and this percentage increases if the child has neurodevelopmental disorders, as autism spectrum (1–3). It is known that even when feeding difficulties are not the main complaint, they will appear during the pediatric evaluation (4, 5). Feeding is more than a mechanical situation, it is a complex system of mechanisms, that involves a comprehensive integrative relation with parents, siblings, caregivers, family, school environment, and cultural habits (1, 5, 6). One of the terms that are being used for this system is Alimentation, referring to all nurturing issues, from access to food to the complex mechanism that regulate the act of feeding inside or outside homes.

In the last years, we have seen a change of the papers regarding feeding difficulties, picky eating or disordered eating, from the initial approach of gastroenterologists, studying organic factors as reflux, esophagitis, failure to thrive and malabsorption issues to a more generalistic approach evaluating the environment and family interaction, as parental styles and habits (7).

The intervention with multidisciplinary team- gastroenterologists, psychiatrists, psychologists, pediatricians, nutrologists, and dietitians– have played an important role, diagnosing feeding difficulties and advising mothers on breast-feeding, postural and physiologic conditions during lactation, allergies, sensorial problems and others (7,8, Machado et al). Integrating teams and with multidisciplinary ways of treating feeding problems have increased understanding, from the diagnosis tools to possible new therapies, using incremental measures, from a simple orientation to desensitization of more complex hyper sensorial children. Nonetheless, sometimes it is not easy to differentiate misperception of caregivers from the real organic causes (1,3–5, 8, Machado et al), showing that the issues in evaluating these children are sometimes and it deserves much more than a superficial approach.

A search in PubMed–March 07th, 2018–using “feeding difficulties” [Title/Abstract] showed 1,506 results. This term, chosen as topic for Frontiers, has already resulted in publications about feeding skills in children with feeding difficulties (Ramos et al), how to evaluate feeding difficulties with scales in children with neurodevelopmental problems (Cavallini et al), and maternal feeding practices in a multidisciplinary research center for this kind of problems (Machado et al).

There is still much to be evaluated, e.g., the need to establish a clinical diagnosis, the mandatory necessity of training pediatricians to observe and tackle this disorder at their offices, and referrals to specialized centers for multidisciplinary treatment when the case is severe.

Some recent findings on this topic showed that children with feeding difficulties usually do not present important clinical issues, but they are within normal range of growth and weight–some of them becoming overweight. The majority of the children do not present important biochemical changes, and they do not present nutritional anemia or nutritional inadequacies as compared to the general population at the same age (Ramos et al). Nonetheless, if they are not carefully and deeply evaluated, and if they are not differentiated from those with secondary issues due to gastrointestinal or allergic disorders, the final result will be a chronic condition with very poor social outcome or will present clinical sequelae.

This could be a conclusion of almost any paper related to childhood diet. Different approaches for the family-child interaction need to be nurtured, and the environment taken into account, to improve feeding as much as possible. Let us continue to study and publish new papers on this wonderful aspect of normal and pathologic conditions of children development.

## Author contributions

MF contributed to the development of feeding difficulties' research topic and drafted this editorial manuscript.

### Conflict of interest statement

The author declares that the research was conducted in the absence of any commercial or financial relationships that could be construed as a potential conflict of interest.
